# ADHD and the female reproductive stages: menstruation, perinatal and menopause

**DOI:** 10.1007/s00737-026-01718-x

**Published:** 2026-05-28

**Authors:** Christine Boyd, Margo Wrigley, Ken Kilbride, Aisling Mulligan, Jessica Bramham

**Affiliations:** 1 School of Psychology, University College Dublin, Dublin, Ireland; 2 Health Service Executive, Dublin, Ireland; 3 ADHD Ireland, Dublin, Ireland; 4 School of Medicine, University College Dublin, Dublin, Ireland

**Keywords:** ADHD, Female health, Women’s health, PMDD, Perinatal, Menopause

## Abstract

**Purpose:**

Gender differences in the presentation of Attention-Deficit/Hyperactivity Disorder (ADHD) are well-documented, yet the interaction between ADHD and female reproductive stages remains underexplored. This study investigates how ADHD impacts menstruation, perinatal experiences, and menopausal symptoms in females.

**Methods:**

A cross-sectional study was conducted with 602 females (mean age = 39.52; SD = 10.21), including 377 with self-reported ADHD and 225 without, recruited through ADHD support groups, social media, Volunteer Ireland, and Prolific. Participants reported their menstrual regularity and completed the Premenstrual Symptoms Screening Tool (PSST). Retrospective postpartum depression levels were measured using the Edinburgh Postnatal Depression Scale (EPDS), and peri- and post-menopausal symptoms were evaluated using the Greene Climacteric Scale (GCS).

**Results:**

Females with ADHD exhibited significantly higher rates of menstrual irregularity (χ2 = 14.2, *p* < .001), more severe premenstrual symptoms (χ2 = 204.7, *p* < .001), elevated postpartum depression levels (t = 7.89, *p* < .001) with increased risk of unplanned pregnancies and pregnancy-related complications, and greater menopausal symptom severity (t = 9.61, *p* < .001) compared to their non-ADHD counterparts.

**Conclusions:**

These findings highlight a higher prevalence of reproductive-related challenges in females with ADHD, underscoring the need for further research in this area and more in-depth analysis. They emphasise the importance of integrating ADHD considerations into female health research and clinical practice.

## Highlights

Females with ADHD experience more severe premenstrual symptoms, including higher rates of PMS and PMDD.

ADHD is linked to higher postpartum depression rates and perinatal complications, underscoring the need for targeted support.

Females with ADHD report more severe climacteric symptoms during menopause.

Further studies addressing reproductive health challenges in females with ADHD is required.

## Introduction

Attention Deficit/Hyperactivity Disorder (ADHD) is a neurodevelopmental condition characterised by inattention, hyperactivity and impulsivity (American Psychiatric Association, 2022 a). It negatively affects executive functioning, including organisation, focus, emotional regulation, and memory (Brown 2009). Adult ADHD prevalence rates range between 2.58% and 6.76% (Song et al. 2021), and recent research suggests nearly equal prevalence in males and females^1^ in adulthood, despite underdiagnosis in females (Hinshaw et al. 2022).

ADHD frequently co-occurs with physical and mental disorders (Kanarik et al. 2022), and there are notable gender differences in these co-occurrences. Females with ADHD are more likely to present with internalised symptoms such as anxiety and depression than males and are more likely to be referred for emotional issues (Vildalen et al. 2019; Williamson and Johnston 2015; Klefsjö et al. 2021). Depression tends to manifest earlier and recur more frequently in females with ADHD compared to those without (Powell et al. 2021). Female sex hormones may influence ADHD presentation, yet research in this area remains sparse, with a recent review identifying only four relevant studies, of which conflicting evidence is presented (Camara et al. 2021). However, growing evidence indicates that ADHD is affected by female reproductive stages, including menstruation, pregnancy, and menopause.

### Premenstrual symptoms

Hormonal fluctuations across the menstrual cycle can cause physical and mental shifts in females (Rehbein et al. 2021). Oestrogen levels drop after ovulation, rise in the mid-luteal phase, and decrease again during menstruation. Declining oestradiol levels (the primary form of oestrogen), may impair executive functioning, including emotion regulation and attention (Eng et al. 2023). Studies have suggested a link between ADHD symptom fluctuation and hormonal changes (Roberts et al. 2018; Haimov-Kochman and Berger 2014). Preliminary findings indicate that adjusting stimulant doses during the premenstrual week may alleviate inattention and mood difficulties (De Jong et al. 2023).

Premenstrual Syndrome (PMS) is recognised as a common condition that is characterised by a range of emotional and physical symptoms including mood swings, anxiety and bloating (Dilbaz and Aksan 2021). Premenstrual Dysphoric Disorder (PMDD) is included in the DSM-5 and involves mood lability, irritability, depression, anxiety, difficulty in maintaining concentration and fatigue, typically emerging for one to two weeks preceding menstruation (American Psychiatric Association, 2022). Females with ADHD are disproportionately affected by PMDD, with prevalence rates as high as 45.5% (Dorani et al. 2021), compared to 1.6% in the general population (Reilly et al. 2024). These findings underscore the need for further investigation into the interaction between ADHD and premenstrual symptoms.

### Perinatal symptoms

A small number of studies suggest that females with ADHD have additional risk factors around pregnancy. They have higher rates of unplanned pregnancies and teenage pregnancies (Meinzer et al. 2020; Owens and Hinshaw 2019) and over half report perinatal complications (Dorani et al. 2021). Females who have had an abortion were three times more likely to report a diagnosis of ADHD (Van Ditzhuijzen et al. 2013). While pregnancy-associated hormonal increases may temporarily mitigate ADHD symptoms (Nadeau and Quinn 2002), postpartum declines in oestrogen and progesterone have been linked to mental health challenges (Trifu, 2019). Postpartum depression (PPD) is elevated in females with ADHD, with reported rates of 57.6%, compared to between 14.5 and 19.6% in females without ADHD (Dorani et al. 2021; Andersson et al. 2023). Andersson et al. (2023) found that 16.8% of females with ADHD were diagnosed with PPD compared to 3.29% in those without ADHD. Given the discrepancy between these prevalence rates, the presence of postpartum depression in the context of ADHD needs further exploration.

### Climacteric symptoms (peri and post-menopausal)

The climacteric period encompasses the perimenopausal, menopausal, and postmenopausal phases, marked by declines in oestrogen and progesterone levels (Greene 2008). Oestrogen and progesterone levels decline during perimenopause and menopause, which affects the release of serotonin and dopamine (Antoniou et al. 2021). As dopamine is a key neurotransmitter associated with ADHD, this additional reduction in dopamine can result in further difficulties with focusing and concentration (Epperson et al. 2015). Evidence suggests that climacteric symptoms, including concentration and mood difficulties, are more severe in females with ADHD (Dorani et al. 2021). However, this life stage remains under-researched in the context of ADHD.

In summary, ADHD in females has been underexplored in clinical and research settings (Young et al. 2020). This study is the first to compare a large sample of females with and without ADHD to investigate the interplay between ADHD and female reproductive stages.

### Research questions

This study aimed to expand upon Dorani et al.’s (2021) work by exploring the following four questions. In comparison with non-ADHD females: (1) Menstruation: Do females with ADHD experience higher rates of PMS and PMDD? (2) Do females with ADHD have higher numbers of unplanned pregnancies, abortions, loss of pregnancy, pregnancy-related complications and higher rates of postpartum depression? (3) Do females with ADHD have more severe menopausal symptoms? (4) Is there a relationship between ADHD symptom severity and severity of PMS, PMDD, PPD and climacteric symptoms?

## Methods

### Participants

**Fig. 1:**
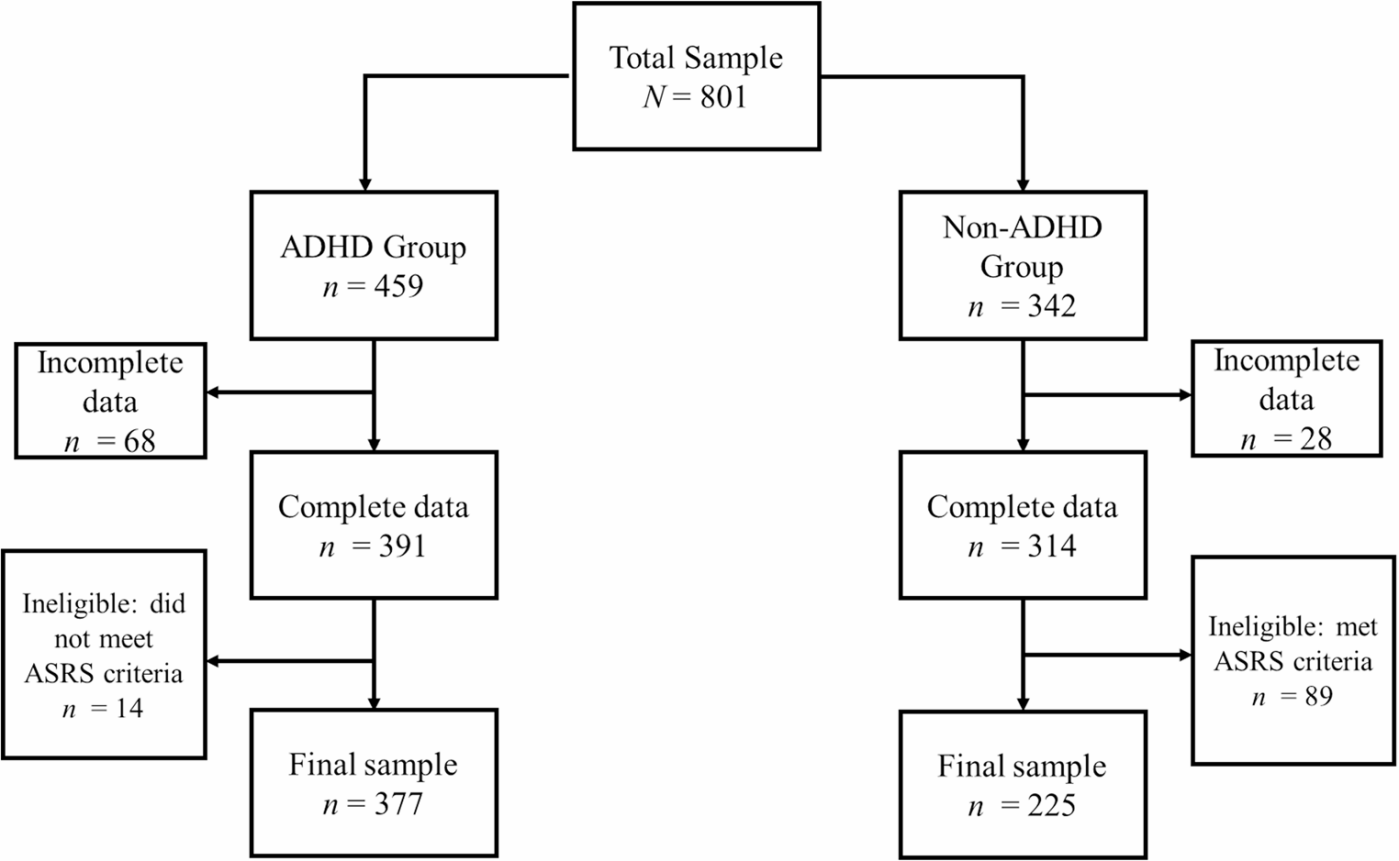
Flowchart of participants

A total of 801 females in Ireland with ADHD participated in this cross-sectional study. Inclusion criteria were females aged 18–69 living in Ireland who had experienced menstruation, pregnancy, or perimenopause/menopause. As shown in Fig. 1, participants were included in the ADHD group if they indicated a diagnosis of ADHD, whether through formal diagnosis or self-identification *and* met the Adult Self Report Scale’s (ASRS) criteria for ADHD (Kessler et al. 2005). Non-ADHD participants did not meet the ASRS criteria. The final sample included 602 females (mean age = 39.52; SD = 10.21), comprising 377 with ADHD and 225 without. ADHD participants were recruited via ADHD Ireland, while non-ADHD participants were recruited through social media, Volunteer Ireland, and Prolific.

### Measures

#### Demographics

Participants provided information on age, education, medications, and medical conditions.

#### Premenstrual symptoms

Menstrual regularity and Premenstrual Symptoms Screening Tool (PSST) responses were collected to assess for PMS and PMDD. The PSST is a 19-item retrospective questionnaire rated on a 4-point Likert scale (0 = not at all, 3 = severe), designed to identify Premenstrual Dysphoric Disorder and Premenstrual Syndrome in line with DSM-IV criteria (Steiner et al. 2003). It shows high sensitivity (79%) in previous studies (Henz et al. 2018) and strong internal reliability in this study (α = 0.95). The scoring for the criteria thresholds are provided in Steiner et al.’s 2003 paper.

#### Perinatal symptoms

Pregnancy history, including number of lifetime pregnancies, number of unplanned pregnancies, miscarriage, stillbirth or abortions, pregnancy-related complications, first pregnancy age and current pregnancy status were collected. PPD was assessed using the Edinburgh Postnatal Depression Scale (EPDS) (Cox et al. 1987), a 10-item scale rated on a four-point Likert scale (0 to 3). The cut-off for PPD is 10 points. The EPDS for this study has high internal reliability (α = 0.93).

#### Climacteric (peri and post-menopausal) symptoms

Participants were asked to report whether they were in the perimenopause stage or post-menopause stage. Climacteric symptoms were assessed using the the Greene Climacteric Scale (GCS) (Greene 2008), a 21-item scale measuring psychological, somatic, vasomotor and sexual dysfunction symptoms, rated on a four-point Likert scale ranging from 0 to 3. The scale has been validated in a number of studies (Greene 2008) internal reliability was high in this study (α = 0.91).

#### ADHD symptoms

ADHD symptoms were assessed using the 18-item Adult Self Report Scale (ASRS v1.1.) (Kessler et al. 2005), a widely used tool for ADHD screening. Items are scored on a five-item Likert scale (1 = never, 5 = very often) and consist of two domains: Part A screens for ADHD, while Part B investigates symptoms. If a minimum of four of the six questions in Part A meet the rating criteria, the symptoms are consistent with an ADHD diagnosis. The ASRS has good concurrent validity (Adler et al. 2006) and high internal reliability in this study (α = 0.96).

### Procedure

Participants completed the measures online via Qualtrics. All participants answered demographic questions and the ASRS. The PSST, EPDS, or GCS were displayed based on their reported health status (menstrual cycle, pregnancy, or menopause).

### Analyses

Data were downloaded to SPSS 29.0 (IBM, Chicago, IL). Percentages, frequencies, ranges, means and standard deviations were used to describe the data. Between-group comparisons were made using the chi-squared test for categorical variables and *t*-tests were used to compare continuous variables. Mann-Whitney tests were used for data that did not follow a normal distribution. Pearson’s correlations were used to examine the relationship between ADHD severity and severity of PMS, PMDD, PPD and climacteric symptoms. The level of statistical significance was set at *p <* .05. Data from 96 participants were removed as they had not completed the ASRS scale and data from 103 were removed as they met the incorrect ASRS criteria levels for their group as per Fig. 1.

## Results

### Demographic

Table 1 shows the age, education status, current medication types and co-occurring conditions for the ADHD group and non-ADHD group.

**Table 1:** General characteristics of the ADHD group and the non-ADHD group

Demographic variable	ADHD *n* = 377	Non-ADHD *n* = 225
Age in years, *M (SD)*	39.50 (9.76)	39.56 (10.95)
Education Level, *n* (%)		
Secondary school up to age 16	6 (1.6)	2 (0.9)
Secondary school beyond age 16	16 (4.2)	6 (2.7)
Post secondary school qualification^a^	80 (21.2)	42 (18.7)
Undergraduate degree	123 (32.6)	61 (27.1)
Postgraduate degree	150 (39.8)	114 (50.7)
Current medication type, *n* (%)		
None	86 (22.8)	136 (60.4)
ADHD medication	152 (40.3)	0 (0)
Antidepressants	120 (31.8)	26 (11.6)
Anti-anxiety medication	65 (17.2)	12 (5.3)
Melatonin	44 (11.7)	4 (1.8)
Hormonal contraceptives	65 (17.2)	42 (18.7)
Other	76 (20.2)	21 (9.3)
Reported medical conditions, *n* (%)		
None	98 (26.0)	154 (68.4)
Mood disorder including depression	133 (35.2)	24 (10.6)
Anxiety disorder	124 (32.9)	23 (10.2)
Personality disorder	7 (1.9)	0 (0.0)
Seasonal Affective Disorder	31 (8.2)	3 (1.3)
Current substance use disorder (alcohol/drugs)	11 (2.9)	1 (0.4)
Substance use disorder in remission	18 (4.8)	3 (1.3)
Delayed Sleep Phase Syndrome	19 (5.0)	0 (0.0)
Hypertension	27 (7.2)	10 (4.4)
Hypothyroidism	34 (9.0)	15 (6.7)
Other	62 (16.4)	11 (4.9)

^a^ Defined as a level 5 or 6 post-Leaving Certificate qualification on the Irish National Framework of Qualifications

An independent samples t-test showed no significant difference in age between the ADHD and non-ADHD groups *t*(600) = 0.1, *p* = .952. Mood disorders including depression (35.2%) and anxiety (32.9%) were reported more frequently in the ADHD group than the non-ADHD group (10.2% and 9.8%). This was also reflected in medication rates, with 31.8% of the ADHD group taking antidepressants compared to 11.6% of the non-ADHD group and more of the ADHD group (17.2%) taking anti-anxiety medication compared to 5.3% of the non-ADHD group.

### Menstrual symptoms

All participants (*N* = 602) completed the menstruation questions and the PSST questionnaire. The results are shown in Table 2.

**Table 2:** Menstrual regularity and PSST scores in ADHD-group (*n* = 377) and non-ADHD group (*n* = 225)

Variable	ADHD *n* = 377	Non-ADHD *n* = 225	Statistic	*p*
Menstrual Regularity, *n* (%)			*χ2 = 14.2*	< 0.001***
Regular menstrual cycle	235 (62.3)	177 (78.7)		
Irregular menstrual cycle	126 (33.4)	45 (20.0)		
Unknown	16 (4.2)	3 (1.3)		
PSST symptoms, *M (SD)*				
Anger/irritability	2.00 (0.80)	1.33 (0.79)	*t* = 9.9	< 0.001***
Anxiety/tension	2.15 (0.80)	1.12 (0.87)	*t* = 14.9	< 0.001***
Tearful	2.27 (0.79)	1.34 (0.96)	*t* = 12.9	< 0.001***
Depressed mood	1.97 (0.93)	1.05 (0.92)	*t* = 11.7	< 0.001***
Decreased interest in home	1.94 (0.89)	0.96 (0.83)	*t* = 13.4	< 0.001***
Decreased interest in work	2.00 (0.89)	0.92 (0.85)	*t* = 14.5	< 0.001***
Decreased interest in social	1.95 (0.92)	1.05 (0.87)	*t* = 11.9	< 0.001***
Difficulty concentrating	2.21 (0.82)	0.80 (0.79)	*t* = 20.7	< 0.001***
Fatigue/lack of energy	2.41 (0.75)	1.55 (0.87)	*t* = 12.7	< 0.001***
Overeating	2.25 (0.86)	1.58 (0.93)	*t* = 8.9	< 0.001***
Insomnia	1.36 (1.06)	0.53 (0.81)	*t* = 10.0	< 0.001***
Hypersomnia	1.61 (0.97)	0.83 (0.88)	*t* = 9.9	< 0.001***
Feeling overwhelmed	2.14 (0.85)	0.97 (0.91)	*t* = 15.9	< 0.001***
Physical symptoms	2.09 (0.84)	1.67 (0.77)	*t* = 6.0	< 0.001***
PSST functionality, *M (SD)*				
Work efficiency	1.92 (0.80)	0.98 (0.82)	*t* = 13.9	< 0.001***
Coworker relationships	1.17 (0.93)	0.46 (0.68)	*t* = 10.1	< 0.001***
Family relationships	1.76 (0.87)	0.91 (0.79)	*t* = 12.0	< 0.001***
Social life activities	1.80 (0.87)	0.95 (0.78)	*t* = 11.9	< 0.001***
Home responsibilities	1.90 (0.86)	0.81 (0.76)	*t* = 15.7	< 0.001***
PMS/PMDD, *n* (%)			*χ2*(2) = 203.98	< 0.001***
No/Mild PMS	50 (13.3)	155 (68.9)		
Moderate to Severe PMS	174 (46.3)	56 (24.9)		
PMDD	152 (40.3)	14 (6.2)		

*p < .05, **p < .01, ***p < .001

The ADHD group had significantly higher frequency of irregular menstrual cycles than the non-ADHD group. Premenstrual symptoms and impact on functionality were significantly higher for females with ADHD across all domains. Rates of PMS and PMDD were significantly higher in the ADHD group than the non-ADHD group.

### Perinatal symptoms

An independent samples t-test showed no significant difference in age between the ADHD group (*n* = 216, *M* = 44.37, *SD* = 7.3) and the non-ADHD group (*n* = 119, *M* = 44.43, *SD* = 9.84) who completed the perinatal questions *t(*333) = 0.1, *p* = .951.

**Table 3:** Perinatal factors and postnatal depression scores in the ADHD-group and non-ADHD group

Variable	ADHD	Non-ADHD	Statistic	*p*
Reproductive status, *n* (%)				
Currently pregnant	4 (1.1)	4 (1.8)	–	–
Ever been pregnant	236 (62.6)	134 (59.6)	*χ2* = 0.6	0.489
Ever had a baby	216 (57.3)	119 (52.9)	*χ2* = 1.1	0.310
Age at first pregnancy, *M (SD)*	28.4 (6.21)	28.61 (5.78)	*t* = 0.3	0.795
Number of babies, *Mdn* (range)	2 (1–6)	2 (1–6)	*U* = 12297.5	0.493
Number of unplanned pregnancies, *Mdn* (range)	0 (0–5)	0 (0–3)	*U* = 13252.5	0.012*
Had a stillbirth^a^, *n* (%)	4 (1.1)	1 (0.44)	–	–
Had a miscarriage, *n* (%)	112 (29.7)	52 (23.1)	*χ2* = 4.5	0.108
Number of miscarriages, *Mdn* (range)	1 (1–9)	1 (1–3)	*U* = 2436.5	0.183
Terminated pregnancy, *n* (%)	46 (12.2)	22 (9.8)	*χ2* = 2.0	0.366
Number of abortions, *Mdn* (range)	1 (1–3)	1 (1–2)	*U* = 480.5	0.991
Pregnancy complications (*n* = 236, 225)				
Antenatal complications, *n* (%)	122 (51.7)	43 (19.1)	*χ2* = 13.3	< 0.001***
Delivery complications, *n* (%)	123 (52.1)	45 (20.0)	*χ2* = 11.8	< 0.001***
Postpartum complications, *n* (%)	112 (47.5)	28 (12.44)	*χ2* = 25.6	< 0.001***
EPDS^b^ total score, *M (SD)*	17.90 (6.76)	11.71 (7.04)	*t* = 7.9	< 0.001***
PPD present^c^, *n* (%)	190 (88.0)	68 (57.1)	*χ2* = 41.2	< 0.001***

*p < .05, **p < .01, ***p < .001

^a^ Chi-square testing was not conducted as the sample size was too small

^b^ Edinburgh Postnatal Depression Scale

^c^ Scored > 10 on the EPDS, meeting criteria for PPD

Table 3 shows the ADHD group had a significantly higher number of unplanned pregnancies, ante, peri and postpartum complications. They had a significantly higher EPDS scores, and a significantly higher number reached the PPD threshold. Although the median number of unplanned pregnancies was 0 for both groups, the ranges were 0–3 and 0–5 for the non-ADHD and ADHD groups respectively, and a Mann-Whitney U Test found significant differences between the two groups. The mean number of unplanned pregnancies were 0.77 for the ADHD group and 0.56 for the non-ADHD group. There were no significant differences between the two groups on childbearing, birth of a child, age at first pregnancy, number of babies, stillbirths, miscarriages, or abortions.

### Climacteric (peri and post-menopausal) symptoms

From the total sample, 218 (36.2%) reported that they were in the peri or postmenopausal stage. A statistically significant difference in age was observed between the ADHD group (*n* = 148; *M* = 48.26, *SD* = 6.01) and the non-ADHD group (*n* = 70; *M* = 51.41, *SD* = 9.06), *t*(216) = 3.0, *p* = .003, indicating that the groups were not fully age-matched.

**Table 4:** Comparison of mean GCS score of peri-menopausal and postmenopausal females (*n* = 218)

GCS domains, M (SD)	ADHD *n* = 148	Non-ADHD *n* = 70	t	*p*
Total score	32.1 (9.99)	18.5 (9.13)	9.614	< 0.001***
Psychological	20.0 (5.66)	10.4 (5.70)	11.662	< 0.001***
Anxiety	10.7 (3.17)	5.4 (3.32)	11.388	< 0.001***
Depression	9.3 (3.13)	5.0 (3.03)	9.531	< 0.001***
Somatic	7.4 (4.36)	4.2 (3.23)	5.499	< 0.001***
Vasomotor	2.7 (1.93)	2.4 (1.94)	1.012	0.313
Sexual Dysfunction	2.0 (1.02)	1.5 (1.07)	3.042	0.003***

*p < .05, **p < .01, ***p < .001

The peri-menopausal and post-menopausal groups were combined and the results are displayed in Table 4. The total score, psychological score and its two subscales anxiety and depression, somatic and sexual dysfunction scores, were significantly higher in the ADHD group than the non-ADHD group. There were no significant differences in vasomotor scores between the groups. The ADHD group reported higher rates (48.6%) of Hormone Replacement Therapy (HRT) than the non-ADHD group (17.1%). HRT did not result in significant differences across the domains in the ADHD group, but it yielded significantly lower rates of anxiety and depression domains in the non-ADHD group.

### Relationship between ADHD symptom severity and menstruation, perinatal and menopausal symptoms

Table 5 shows the relationships for the whole sample and the ADHD and non-ADHD groups separately. Pearson’s correlations were conducted to examine the relationships between ADHD severity, as measured by the total score on the ASRS symptoms, and the total symptom scores for the PSST, EPDS, and GCS.

**Table 5:** Correlation between ADHD Severity and Reproductive Stage Variables

Reproductive variable with ASRS total	ASRS total symptom score		
	Total	ADHD	Non-ADHD
Premenstrual	*n* = 600 *r* = .69**	*n* = 376 *r* = .40**	*n* = 224 *r* = .40**
Postpartum	*n* = 335 *r* = .51**	*n* = 216 *r* = .35**	*n* = 119 *r* = .42**
Menopause	*n* = 218 *r* = .64**	*n* = 148 *r* = .47**	*n* = 70 *r* = .26*

*p < .05

**p < .001

ADHD symptom severity was correlated significantly with premenstrual total symptoms, postpartum depression total and menopause symptoms for the total sample and for the ADHD and non-ADHD groups when analysed separately.

## Discussion

This study aimed to determine whether females with ADHD experience greater challenges than females without ADHD regarding menstruation, the perinatal period, and menopause. It further explored whether there is a relationship between ADHD symptom severity and severity of PMS, PMDD, PPD and climacteric symptoms. Given the cross-sectional design of the study, causality cannot be inferred.

### Menstrual symptoms

The results highlight significant differences between the ADHD and non-ADHD groups concerning menstrual regularity, premenstrual symptoms, PMS, and PMDD rates, as well as the impact of these symptoms on functionality. Significantly elevated rates of PMS and PMDD were found among females with ADHD compared to those without ADHD. Specifically, the prevalence of moderate to severe PMS and PMDD was substantially higher in the ADHD group, indicating a heightened susceptibility to severe premenstrual symptomatology and its associated challenges among females with ADHD.

Moreover, findings show a substantial disparity in premenstrual symptomatology between the two groups. Females with ADHD reported significantly higher symptoms across all domains when symptoms compared to their non-ADHD counterparts. These results suggest that ADHD exacerbates premenstrual symptoms, contributing to greater emotional and physical distress during the menstrual cycle for females with ADHD. Additionally, the impact of premenstrual symptoms on functionality was markedly higher among females with ADHD across all domains assessed, underscoring the substantial burden that premenstrual symptoms impose on the daily functioning and interpersonal relationships of females with ADHD, highlighting the need for targeted supports to address these challenges comprehensively.

The ADHD group exhibited a notably higher frequency of irregular menstrual cycles compared to the non-ADHD group, suggesting an association between ADHD and menstrual irregularity. This finding closely corresponds to previously documented rates (Dorani et al. 2021), emphasising the importance of considering menstrual health as a component of comprehensive care for females with ADHD.

This is the first study to compare premenstrual symptoms in females with ADHD and without, emphasising the importance of recognising and addressing menstrual health issues among females with ADHD. Further research is needed to explore the underlying reasons for these findings and to assess the availability, utilisation, and adequacy of supports for females with ADHD with premenstrual issues in Ireland.

### Perinatal

The perinatal results provide insights into the association between ADHD and perinatal challenges, as well as their impact on PPD scores. The ADHD group had significantly higher postpartum depression scores and rates compared to the non-ADHD group. These findings highlight the elevated risk of PPD among females with ADHD in Ireland, emphasising the importance of early identification and intervention to mitigate adverse effects on maternal and child well-being. This study is the first to explore this topic within an Irish context, potentially informing perinatal care and supporting the education of females about PPD risk factors prior to pregnancy.

While the median number of unplanned pregnancies was the same for both groups, the ADHD group reported a significantly higher range, suggesting a greater incidence of unplanned pregnancies. Menstrual irregularities and challenges with consistent contraception use, due to ADHD-related difficulties with executive functioning, may contribute to this heightened rate (Lundin et al. 2023; Shoham et al. 2021).

No significant differences were found between the groups regarding age at first pregnancy, the number of abortions, or negative reproductive outcomes such as stillbirths or miscarriages. However, females in the ADHD group reported a significantly higher frequency of antenatal, perinatal, and postpartum complications compared to those in the non-ADHD group, aligning with previous research (Dorani et al. 2021; Walsh et al. 2022). The study also identified significantly higher rates of unplanned pregnancies among females with ADHD. Collectively, these findings emphasise the importance of integrated, ADHD-informed perinatal care pathways. Clinicians should consider ADHD as a potential risk factor during reproductive planning and pregnancy.

### Perimenopause/post menopause

The ADHD group reported greater climacteric symptoms overall, including higher psychological, anxiety and depression scores. This aligns with the established link between ADHD, anxiety, and depression (Vildalen et al. 2019; Williamson and Johnston 2015). While vasomotor scores were slightly higher in the ADHD group, no significant differences were identified, contrary to previous research. Other factors such as BMI and levels of exercise may influence these results (Gold et al. 2017; Liu et al. 2022).

Notably, the ADHD group reported 2.8 times higher rates of HRT usage than their non-ADHD counterparts. This may reflect more severe climacteric symptoms in the ADHD group or misinterpretation by clinicians of pre-existing ADHD difficulties as menopause-related. Further research would clarify whether HRT interventions are effective for females with ADHD and how best to address overlapping symptoms.

No difference was observed between the two groups regarding sexual dysfunction, suggesting that hormonal changes impact females with and without ADHD similarly. However, the correlation between severe ADHD symptoms and climacteric symptoms should be considered when supporting females with ADHD during this life stage. This study is the first to explore ADHD symptoms during the climacteric stage in females in Ireland, providing valuable insights into the challenges faced by this population.

### Limitations

Several limitations should be acknowledged. Firstly, older participants were asked to retrospectively report their menstrual experiences, which introduces the possibility of recall bias. However, menstrual symptoms represent recurrent and often clinically significant experiences across many years of the reproductive lifespan, and their inclusion allows for representation of cumulative reproductive experiences. Future research could adopt longitudinal designs to more precisely capture symptom trajectories. Secondly, while participants met ASRS criteria for ADHD, self-selection may have introduced bias, as individuals could meet the criteria due to other mental health conditions. As such, findings should be interpreted with precaution. Future research could refine this research by recruiting participants with a formal ADHD diagnosis. Thirdly, PPD was assessed only after the birth of the first baby, potentially overlooking cases arising with subsequent births. Next, the peri/post menopause group was not fully age-matched, with the non-ADHD group being significantly older. As menopausal symptom severity may vary with age and stage progression, age may represent a potential confounding factor in the observed group differences. The ADHD group reported higher use of medications which may have influenced mood, sleep, and cognitive symptoms, potentially confounding symptom reports. Sensitivity analysis adjusting for medication use and age would be informative in future work. Finally, this study did not collect data on race, ethnicity, or socioeconomic variables which may limit external validity.

## Conclusion

This study provides important insights into the challenges faced by females with ADHD across key reproductive stages, including menstruation, the perinatal period, and menopause, within the Irish healthcare context. These results support the need for routine enquiry regarding menstrual health, pregnancy planning, postpartum mood, and menopausal symptoms in females with ADHD. Similarly, health providers should consider ADHD symptomatology when assessing cyclical mood changes, perinatal distress, and climacteric difficulties in females.

An integrated, ADHD-informed approach to female healthcare may facilitate earlier identification of vulnerability, improve reproductive planning support, and enable timely psychological or pharmacological intervention during high-risk periods such as the postpartum phase and perimenopause. The findings highlight the importance of lifespan-oriented, gender-sensitive ADHD care. Future research should further refine screening pathways and treatment protocols to better support females with ADHD across reproductive transitions, ultimately improving maternal health outcomes and overall quality of life.

## Footnotes

1 Numerous research papers have used the terms “female” and “women” interchangeably and have not asked participants to specify their sex. We recognise that there are a range of gender identities who were assigned female at birth (AFAB). To enhance clarity, this study considers menstruation, pregnancy and menopause which are stages relevant to the female reproductive lifecycles, so we refer to sex (female) rather than gender identity (woman). In reporting previous research, sex and gender usage will be reported transparently (Heidari et al. 2016).
